# 

**Published:** 1892-02-06

**Authors:** 


PRICE ONE PENNY.

No. 280, Vol. XI.—February 6th, 1892.

[Registered at the General Post Office as a Newspaper for transmission in the United Kingdom and Abroad.]

WITH EXTRA NURSING SUPPLEMENT.

Per Annum, 6s. 6d., post free.

Monthly Parts, 6d.; post free, 8d.

Ditto per Annum, 8s., post free

### THE HOSPITAL

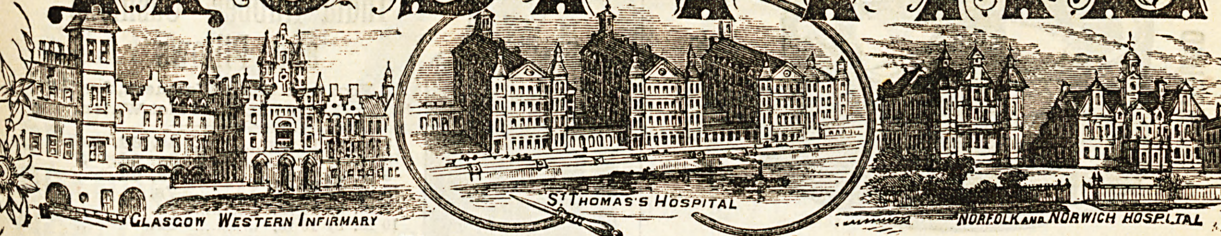

A WEEKLY JOURNAL OF

Science, Medicine, Nursing, and Philanthropy.

SATURDAY, FEBRUARY 6th, 1892.

**The Stable Door and the Stolen Steed** . . . . 225

**Passing Topics.**—Pegging Away . . . . 226

**Some Scientific Aspects of Insanity.** XI.—Epileptics . 227

**Some American Hospitals.** VI.—The Philadelphia Hospital, Blockley . . . . 228

**Letters from Far Japan.** By Mrs. Ernest Hart. II.—Hospitals and Medical Education in Japan. Part I. . . 229

**Everybody's Column** . . . . 229

**Notes and News** . . . . 230

**General Charities** . . . . 231

**Scraps and Gleanings** . . . . 231

**Annotations.**—Mr. Spurgeon—Lady Smokers Again—Religious Foolishness . . . . 232

**The Practitioner's Mirror and Retrospect—**

Medicine.—Gastric Dyspepsia . . . . 233

Ophthalmology.—Eye Complications in Influenza . . 234

Practitioner's Bookshelf.—Papers on Physical Education 234

New Drugs, Appliances, and Things Medical.—The "Humane" Seat—Swinborne's Isinglass . . . 235

**The Hospital Sunday Fund** . . . . 235

**The Story of the Insane from Year to Year** . . 236

**The Student of Medicine.**—Appointments—Vacancies.

EXTRA SUPPLEMENT.—THE NURSING MIRROR.

**En Passant.**—Perth Nursing Society—Buchanan Cottage Hospital—A Lesson for the Public—Short Items—Sheffield Workhouse Hospital—Glasgow Nurses . . . cix

**A Canadian Training School** . . . . cx

**On the Nursing of Children.** II.—Emergencies (*continued*) cx

**County of Cornwall Trained Nurses' Home** . . cxi

**For Reading to the Sick.**—Irritability . . . cxi

**Our Indian Letter** . . . . cxii

**Be not Weary** . . . . cxii

**Everybody's Opinion.**—Nursing at West Cowes—An Appeal to the Benevolent—Brentford District Schools . . cxiii

**Death in Our Ranks** . . . . cxiii

**Presentations—Appointment** . . . . cxiii

**"Sister is Wanted"** . . . . cxiii

**Notes and Queries** . . . . cxiii

**Off Duty.**—The Blind Man's Dog . . . . cxiv

